# Mechanism-informed transition from pediatric to adult care in sickle cell disease: a case study

**DOI:** 10.1186/s12913-026-14312-9

**Published:** 2026-03-13

**Authors:** Jane S. Hankins, Tarun Aurora, Rachel Matsumoto, Sara Malone, Ana A. Baumann

**Affiliations:** 1 Department of Hematology, St. Jude Children’s Research Hospital, Memphis, TN USA; 2 Department of Global Pediatric Medicine, St. Jude Children’s Research Hospital, Memphis, TN USA; 3 Department of Hematology and Medical Oncology, Emory University, Atlanta, GA USA; 4 School of Public Health, Washington University in St. Louis, St. Louis, MO USA; 5 Division of Public Health Sciences, Department of Surgery, Washington University in St Louis, St. Louis, MO USA

**Keywords:** Sickle cell anemia, Causal pathway diagram, Causal loop diagram, Health care transition, 6 core elements of health care transition, Adult care transition

## Abstract

**Background:**

Healthcare transition (HCT) refers to the shift from a child-centered to an adult-centered model of care, a process that, when well-functioning, can reduce disengagement and improve satisfaction with care. The Six Core Elements (6CE) framework structures HCT services; however, its implementation process remains understudied, limiting its broader applicability. Learning how to effectively implement the 6CE strategies can enhance their application in real clinical contexts. This case study describes the successful implementation of a 6CE-informed transition program for adolescents and young adults with sickle cell disease (SCD), a genetic condition associated with reduced life expectancy. We also propose a mechanism-informed approach to guide future HCT implementation.

**Methods:**

We created a matrix that mapped the transition program activities and domains to the 6CE. Next, we retrospectively created an implementation research logic model. We used it to build causal pathway diagrams that outlined and mapped the contextual determinants to the implementation strategies and their purported mechanisms. To understand the dynamics of each implementation strategy, we built expert consensus and created a causal loop diagram (CLD) that illustrated the interrelationships among the strategies.

**Results:**

Four main strategies were identified, each operated across multiple levels and via different mechanisms: (1) Organizational directives worked through rule-setting to increase mandates regarding HCT, (2) SCD transition team worked through care structuring at the organizational level and increased empowerment at the provider level, (3) Monitoring and evaluation enacted HCT activities through increased goals and planning at the organizational level and action planning at the provider level, and (4) Care co-location activated information exchange between pediatric and adult institutions and enhanced influence in decision-making among providers. CLDs identified dynamic variables of the context that impacted the effect of the strategies and uncovered three key underlying processes necessary to implement and likely maintain the 6CE implementation: capacity development, collaboration, and leadership buy-in.

**Conclusion:**

This study identified implementation strategies that facilitated the adoption of the 6CE within a SCD transition program and the mechanisms involved. We developed a mechanism-based HCT model for future empirical testing and refinement, aiming to enhance the transportability and adaptability of HCT services across diverse healthcare settings.

**Supplementary Information:**

The online version contains supplementary material available at 10.1186/s12913-026-14312-9.

## Contributions to literature

Healthcare transition (HCT) frameworks enhance health outcomes for adolescents and young adults; however, their implementation remains challenging due to the multifaceted nature of their components and the multi-level targets they address.

Optimizing HCT implementation requires a deeper understanding of how implementation strategies function and produce outcomes.

Using a sequential approach that combined causal pathways and causal loop diagrams, we retrospectively identified four key implementation strategies and elucidated their underlying mechanisms.

We propose a mechanism-informed model for implementing the Six Core Elements of HCT, detailing how implementation strategies contribute to the successful implementation of these elements.

**Supplementary Information:**

The online version contains supplementary material available at 10.1186/s12913-026-14312-9.

## Background

Sickle cell disease (SCD) is one of the most common inherited genetic hematologic conditions worldwide. SCD was once considered a childhood disease with high mortality during early childhood, and only about 50% of those affected survived to age 18 in the 1970s [1]. The adoption of evidence-based interventions such as birth diagnosis, infection prevention (e.g., prophylactic penicillin, anti-pneumococcal vaccines), and hydroxyurea therapy has progressively curtailed pediatric SCD mortality over the past 5 decades [2, 3]. As a result, almost all children with SCD survive to age 18 in settings where adequate access to evidence-based treatments exists [2, 4]. However, survival decreases substantially in early adulthood, with only about 80% of individuals with SCD in the United States living to age 30 [5, 6]. Between the ages of 18 and 35, individuals with SCD experience an increase in acute disease complications, leading to more frequent use of acute healthcare services [7]. Health deteriorates between adolescence and young adulthood, as emerging adults face adverse health outcomes exacerbated by the complexity of transitioning from pediatric to adult care, underscoring the need for healthcare-directed interventions during this vulnerable period.

### The sickle cell disease healthcare transition gap

Health care transition (HCT) is the process of moving from a child-centered to an adult-centered model of health care [8]. HCT has 3 main activities: transition planning, transfer of care, and integration into adult care [9]. HCT of pediatric patients with chronic medical illnesses, such as SCD, is complex due to adolescents’ multiple medical, psychosocial, and educational needs that increase in frequency as they age into adulthood [10, 11]. For instance, prevalent cognitive dysfunction places youth with SCD at greater risk of low educational achievement and impaired instrumental activities of daily living [12–14], highlighting the need for support with navigating changes during HCT. Despite its importance, the transition from pediatric to adult care is often poorly planned or non-existent [15]. The fragmented transition process leads to lower adherence to medication [16], increases in acute care utilization [11], worsened mental health [10, 17], greater disease severity [18], and premature death [19]. While no universally established model for a transition program exists, there are recommendations to ensure adult care transition [20–27]. These recommendations offer a framework to structure patient-centered activities during youth planning, transfer, and integration into adult care.

### A structured approach for health care transition

The GotTransition™ Six Core Elements of HCT (6CE) is a framework designed to structure and support the transition of youth with chronic diseases from pediatric to adult care [28]. The 6CE are designed to guide and support youth in establishing adult health care practices and outline the following elements of a structured transition process: (1) transition policy (2), tracking and monitoring progress (3), assessment of transition readiness (4), planning for adult care (5), transferring to adult care, and (6) integration into adult care [28]. The 6CE has been widely used to transition care for patients with chronic medical conditions [29]. Data from institutions that have implemented structured programs for transitioning patients to adult care indicate positive results, including improved medication adherence and self-care management skills, increased patient satisfaction with care, fewer barriers to accessing care, reduced gap between pediatric and adult care, and decreased hospitalizations [30, 31]. In SCD, using the 6CE has increased early engagement in adult care, decreased pediatric care abandonment, and increased adult care retention [32–34]. In 2018, the American Academy of Pediatrics, the American Academy of Family Physicians, and the American College of Physicians jointly endorsed the use of the 6CE [28]. 

Although many institutions have utilized the 6CE for different conditions, including SCD [29], its customization and full applicability have not been widely adopted [9]. One of the major obstacles to the wider use of the 6CE is the lack of guidance on how to implement it and how to adapt the framework to different diseases and contexts, particularly those in low-resource settings. Although quality improvement methods have been successfully used to implement all 6CE, or some of them [35, 36], they do not explicitly outline the process of selecting context-specific strategies to implement the 6CE.

### The complex process of implementing HCT

Part of the challenge in identifying the tools needed to implement the 6CE is their lack of operational definitions [37]. Because HCT programs are complex, multi-level, interrelated, and span several years (typically from ages 12 to 23), strategies to implement them are also complex. As a result, there is variability in how 6CE are implemented in different settings, thus resulting in inconsistency in their clinical effectiveness and difficulties in identifying what works for whom, when, and how, thus impairing scientific reproducibility and testing their effectiveness [38–40]. 

### Implementation strategies are central to implementing HCT

Implementation strategies are the processes used to enhance the adoption, implementation, and sustainability of evidence-based clinical practice, guidelines, or programs [41]. Most HCT programs have used implementation strategies without properly evaluating their mechanisms of action. To understand how to implement the 6CE, we need clarity regarding the function or mechanisms of the implementation strategies, their relationships with the efficacy of the intervention (i.e., the 6CE), and the context in which they are being implemented. Mechanisms are processes through which an implementation strategy operates to affect a desired implementation outcome [42]. Researchers have used various forms of causal mapping to outline the mechanisms of action of strategies, including causal pathway diagrams and causal loop diagrams (CLDs). Causal pathway diagrams are a way to outline the causal mechanisms that explain how a particular strategy works and outline (1) the specification of the strategies (2), the strategy-mechanism linkage (3), the proximal and distal outcomes, and (4) articulate the effect modifiers [43]. CLDs, originating from system dynamics, are tools that visually represent how variables are interconnected to form a complex system [44]. CLDs describe the relationships between variables, emphasize interconnectedness, and provide a dynamic, non-linear depiction of relationships within a complex system [45]. The goal of causal maps, which depict areas of feedback within systems, is to make explicit the assumptions of each strategy. This leads to a more comprehensive understanding of the implementation of an evidence-based treatment or program (in our case, the 6CE) and subsequently facilitates better testing of the implementation process.

### The St. Jude pediatric-to-adult care program for SCD

In 2007, St. Jude Children’s Research Hospital (St. Jude) initiated a transition to adult care program for youth with SCD (hereafter referred to as St. Jude HCT Program), progressively built based on consensus statements and recommendations from professional societies [25, 46]. The St. Jude HCT Program supports adolescents and young adults with SCD in planning, transferring, and integrating into adult care [32, 47]. The program offers comprehensive training and health education for patients, including both in-person and online sessions focused on disease literacy [48, 49], academic planning, and personal health history [50]. It also provides experiential learning opportunities to build transition skills, such as hands-on training in appointment scheduling, medication refills, understanding insurance benefits, and early exposure to adult healthcare facilities [51, 52]. Neurocognitive and readiness assessments are conducted periodically [53–55] and used to tailor transition planning [56]. Transition barriers unique to each patient are identified through transdisciplinary activities, and individualized transition plans are devised [57]. Patients transfer to adult care at 18 or when deemed transition-ready. Integration into adult care is provided until age 25 at one of two adult partner centers [58]. In this program, 82% of young adults initiate adult care, and 75% remain after 2 years [33, 59]. Additionally, health-related quality of life among young adults with SCD was positively associated with participation in a greater number of disease-literacy–building sessions [59]. For this study, we use the St Jude HCT Program as a case study to develop the hypothesis of the mechanisms for the strategies to implement the 6CE. This study aims to retrospectively describe implementation strategies for the 6CE in SCD within one transition program setting and hypothesize the mechanisms of function for these strategies to facilitate future 6CE implementation in diverse settings and populations.

## Methods

### Summary of activities

We engaged in a four-step process for this work: (1) specified the HCT activities, (2) mapped the determinants to implementation strategies, (3) isolated the causal pathway for each implementation strategy, and (4) combined all identified mechanisms in a causal loop diagram map. This culminated in a diagrammed model for the mechanisms of action of the main strategies used to implement HCT for SCD in the St. Jude HCT Program. Below, we describe the steps.

### Step 1. Specification of HCT activities

First, the second author (T.A.) conducted a series of in-depth discussions with the first author (J.S.H.), the founding director of the HCT program, to recount the program’s creation and review all program activities and procedures. Next, to specify the HCT activities and understand how the St. Jude program implemented them, T.A. employed participant observation. Participant observation is an ethnographic method in which an observer immerses themselves in a particular social setting or group, observing the participants’ behaviors, interactions, and practices [60]. Information regarding HCT staff member procedures regarding both the method and timing of program activity was gathered by T.A., including any materials and tools used (e.g., education tools). This involved attending team meetings and immersing himself within the clinical environment, making silent observations of provider-patient interactions and clinical work during clinic visits that involved care transitions. Each program activity and procedure was then listed using the Template for Intervention Description and Replication (TIDieR) guide [61] (TIDieR Supplementary material). A matrix of intentions, authors, process, and locations was created, specifying HCT activities at the patient, provider, and institutional levels, and mapped to each 6CE. The matrix was then discussed with the transition team.

### Step 2. Development of the program’s logic model

We created an implementation research logic model (IRLM) to understand the connections between determinants, strategies, mechanisms, and outcomes [62]. We first identified the determinants (barriers and facilitators) to implementing the 6CE in the St. Jude HCT Program and then mapped these contextual determinants to the implementation strategies. Determinants were identified through literature review, needs assessment research conducted during the development and implementation of the St. Jude HCT Program [51, 63, 64], and team discussions. We mapped each program activity within the 6CE to the determinants and the implementation strategies and classified the strategies according to the Expert Recommendation for Implementing Change (ERIC) [65, 66], which facilitated the identification of corresponding theories. The determinants and components of each implementation strategy were identified through iterative consensus discussions with the hospital’s clinical staff, patient advocates, program members, and community adult providers. We then hypothesized the mechanisms whereby each strategy functioned in relation to the program’s determinants. We adopted the common definition of mechanisms in Implementation Science, which is conceptualized as the functions of the strategy that explain how and why it works to address a determinant [43]. Theories are often underutilized in the development of implementation strategies, impairing their testing and study replicability [41, 67, 68]. Thus, as a key step, we considered theories to help explain how and when determinants influence the implementation process and the mechanism of the strategies [43, 69]. 

### Step 3. Isolate the mechanism of each implementation strategy

Data within the IRLM were organized into variables and conditions within domains (i.e., inner setting, outer setting) that depicted how each HCT activity fit together to produce the adoption of HCT. We used the Taxonomy of Behavior Change Methods and the Behavior Change Technique Taxonomy [70, 71] to classify the behavioral change promoted by the hypothesized strategies’ mechanisms. From the IRLM hypothesized mechanism, we then built individual causal pathway diagrams for each strategy, incorporating information from the literature related to the purported function of a strategy and the contextual factors (determinants of implementation) identified in Step 2. We conducted member checking with the clinical staff, patient advocates, and program members during the monthly team meetings to review the plausibility of the causal pathway diagrams and refine them through consensus.

### Step 4. Combine the mechanisms of all implementation strategies

In the final step, we reviewed all prior steps, synthesized content into one CLD, and highlighted specific reinforcing and balancing loops within the main CLD. This diagram illustrates the underlying structures of SCD transitional care, highlighting the various mechanisms and sources of feedback that work together to explain the system dynamics within the St. Jude HCT program. Our CLD includes the previously described implementation strategies and mechanisms and their relationship to the identified outcomes [43, 72]. Different components of the 6CE are also visualized within the CLD. This step extends the prior work on mechanisms (e.g., causal pathway diagram) by visually depicting aspects of the system that the interventions (i.e., 6CE) will change and impact. This work did not meet the definition of research, as defined in 45CFR46.102.

## Results

### Step 1. Specification of HCT activities

Table 1 shows the determinants, the implementation strategies (along with their specifications) within the St. Jude HCT Program. Here, we define *organizational directives* as directions created by the transition team to specify when to initiate the HCT process, when to transition to adult care, and the steps required to achieve the HCT process. *The SCD transition team* is a new care team, a dedicated group of individuals willing to take on new roles related to HCT to implement patient-centered interventions in accordance with the 6CE and monitor program activities for quality assurance. *Monitoring and evaluation* are defined as tracking the patient’s progress in the HCT milestones (monitoring) and synthesizing data to inform policy and program development (evaluation) of the HCT program. Finally, *care co-location* involves integrating pediatric and adult care into the same facility to support HCT services.

**Table 1:** Determinants and the implementation strategies developed for the St Jude HCT Program

Determinant	Implementation Strategy	Definition	Action	Actor (who delivers the strategy)	Action Target	Dose (frequency)	Outcomes	Theories informing
Lack of mandate or guidance regarding the timing of initiation of HCT services (i.e., the timing of transfer and transition preparation), the process of HCT services, and guidance about transfer metrics.	Organizational Directive	Clear institutional guidance outlining when to begin the process of transition preparation, when to transfer to adult care, and how to prepare patients and caregivers for adult care. A clear outline of roles in case the hospital has pediatricians and adult providers in the same organization, or there is no change in provider.	Transition policy used by providers	Organizational leadership	Transition team	Once	Adherence to SCD transition policies	Institutional Theory
Skilled staff not delivering HCT services No structure to deliver HCT services	SCD transition team	Development of a new team to assist adolescents and young adults with SCD transition from pediatric to adult care. Outline roles and skills of each clinical team member, adding different disciplines and different skills as needed.	One team member becomes the team agent to advocate for resources and communicate with leadership. Existing staff in the institution add new roles/shift roles to deliver HCT services to their existing clinical duties, and one staff member becomes the transition coordinator.	Entire new transition team	Providers	Continuous	Adoption of HCT practices	Institutional theory Institutional logics Professions and organizations and social identity [73]
Lack of structure for identifying eligible patients to receive HCT services Lack of a process to monitor the progress of patients Lack of a process to monitor the progress of the program	Monitoring and Evaluation (M&E)	Develop and organize systems and procedures that monitor clinical processes and outcomes for quality assurance and improvement.	Collect data, input, and analyze outcomes at the program and patient levels.	Entire new transition team	Providers	Continuous	Adoption of HCT practices	Social network theory [74, 75] Monitoring and evaluation [76–78] Audit and Feedback [79, 80]
Care fragmentation (due to lack of care coordination) Care discontinuity (when changing providers) Lack of communication between pediatric and adult providers	Care co-location (pediatric and adult care)	Structural change in healthcare provision or an organizational strategy in which providers, pediatric and adult, integrate care and work together in the same facility	Pediatric & adult providers work in the same facility and co-manage (share) the care of the same patients. They share patient information within their respective disciplines (expertise) and devise common care pathways.	Pediatric and adult healthcare providers	Providers	Twice per month	Adoption of HCT practices Care coordination Care integration Development of interpersonal, collaborative relationships	Care coordination literature Network theories [81] Transaction cost theory [82] and resource-dependent theory [83] Professions and organizations, and social identity

The observer (T.A.) spent a total of four clinic days observing the transition clinic activities; he observed a total of fifteen patient-provider interactions of six key providers from the St. Jude HCT Program (one hematologist, one social worker, two nurse care coordinators, and two clinic nurses). Table 2 shows the matrix of the observed program activities mapped to each 6CE: the core element of the framework, the implementation strategy identified in the St. Jude HCT Program to support each 6CE, the description of the HCT activities, and the description of the activities at the staff and organizational level as well as at the patient and caregiver levels based on the participant observation. In some instances, implementation strategies enacted several 6CE simultaneously to promote different activities within the program. For instance, the strategy SCD transition team enacted Core Elements 3 and 4, transition readiness assessments (applying the transition assessment tool), and transition planning (disease education), respectively (Table 2).

**Table 2:** Description of the 6CE and their implementation strategies

Six Core Elements of Health Care Transition (HCT)	Implementation Strategy	Components of the HCT activities	Description of HCT activity	
			Staff and organizational levels	Patient and caregiver levels
Core Element 1: Transition Policy	SCD transition team	1. Team leader (advocate agent) 2. Team coordinator 3. Other staff willing to fulfill roles related to HCT 4. Forum for regular engagement and idea generation 5. Creation of policy and guides to execute work	The leader (advocate agent) mobilizes resources and engages the leadership. The team coordinator organizes activities and develops standard operating procedures to operationalize HCT activities. Both the team leader and the coordinator ensure team cohesiveness and maintenance of engagement by reviewing challenges, celebrating successes, and welcoming new ideas. Other team members participate in team meetings, jointly make decisions, and deliberate and create tools (e.g., transition policies) to execute work.	Engagement of patients and caregivers in the HCT process: Patients are prepared to be active in their care, to ask questions, and specifically to inquire about care guidelines, the evidence behind clinical decisions, or available evidence-supported treatments.
Core Element 1: Transition Policy	Organizational Directives	Clear guidance on when to start the transition process to adult care (e.g., age of patient) and when to transfer patient (e.g., age and readiness level) to adult care. Clear guidance on how to prepare for transfer (e.g., Six Core Elements).	Directives from the SCD care team outline the decision trees for the clinical team to follow.	Transition directives are presented to patients and their caregivers, and the content is explained: The transition team informs patients and their caregivers when the transition process will begin, what it will consist of, and when the transfer will occur.
Core Element 2: Tracking & Monitoring	Monitoring and evaluation	1. Utilize software to create a transition database and tracking system. 2. Create a list of variables and define their frequency of documentation. 3. Define who documents, when, and how. Define who will provide the reports to whom, when, and how. 4. Monitor the quality and quantity of imputed data.	Development of a database for tracking the program’s data used by team members to input data from eligible patients. Team members use data to identify eligible patients to start the transition process, track individual patients’ progress, and aggregate data to inform program-level progress. Data, along with organizational policy, is used to inform the timeline of transfer of care.	n/a
Core Element 3: Transition Readiness	Monitoring and evaluation	Monitor quality metrics and evaluation of patients’ and program outcomes for goal-setting.	Use an assessment tool (e.g., the Self-Management Skills Checklist (48, 53)) to evaluate the patient and caregiver’s readiness for transition.	n/a
	SCD transition team	Team members use the transition readiness assessment to collect patient and caregiver data.	Staff perform data collection and data imputation. The transition team uses readiness data and clinical judgment to tailor transition planning and preparation.	Adolescents and caregivers complete readiness assessments, and the results are used to tailor their transition preparation.
Core Element 4: Transition Planning	Monitoring and evaluation	Monitor quality metrics and evaluation of patients’ and program outcomes for goal-setting.	The transition team discusses patient readiness assessments and medical history to reach a consensus on each patient’s individualized transition plan. Aggregate data is used to monitor and evaluate program progress and guide future modifications for improvement.	n/a
	SCD transition team.	1. Team members assume new roles and add patient teaching responsibilities, such as teaching patients about self-care, which takes place both in the pediatric and adult settings.	Team members provide disease education and transition skill-building to eligible patients.	Patient and caregiver educational and skill-building sessions. Educational and training material was developed and used during in-clinic and online educational sessions. Experiential learning sessions train patients to gain transition skills.
Core Element 5: Transfer of Care	SCD transition team	1. Team members help patients choose adult providers by introducing options before leaving pediatric care. 2. Team members develop a transfer summary. 3. Team members schedule the first adult visit with the provider of the patient’s choice.	The transition team identifies patients who are approaching transfer, facilitates the introduction of adult providers, and ensures patient summaries are sent to new providers using transfer notes.	Early introduction to adult care: Patients and their caregivers are introduced to adult care before they leave pediatric care (e.g., “Transition Tour [52]”), have their first appointments scheduled, and are informed about their upcoming appointments.
		The transition team makes referrals to accepting adult providers. Adult providers accept patients. Team members send patient summaries to the next provider	Adult providers accept the responsibility of caring for the transferred patients. The transition team ensures that patients’ data reaches adult providers.	n/a
Core Element 6: Transfer Completion (adult care integration)	Care co-location (pediatric and adult care)	Co-location of pediatric and adult providers in the same facility. Pediatric and adult providers see patients during the same visit. Pediatric and adult providers agree on a plan for eligible patients	Pediatric and adult providers work together in the same physical space to integrate patients into adult care. Both see the same patients and devise a care pathway together.	n/a

### Step 2. Development of the program’s logic model

**Fig. 1:**
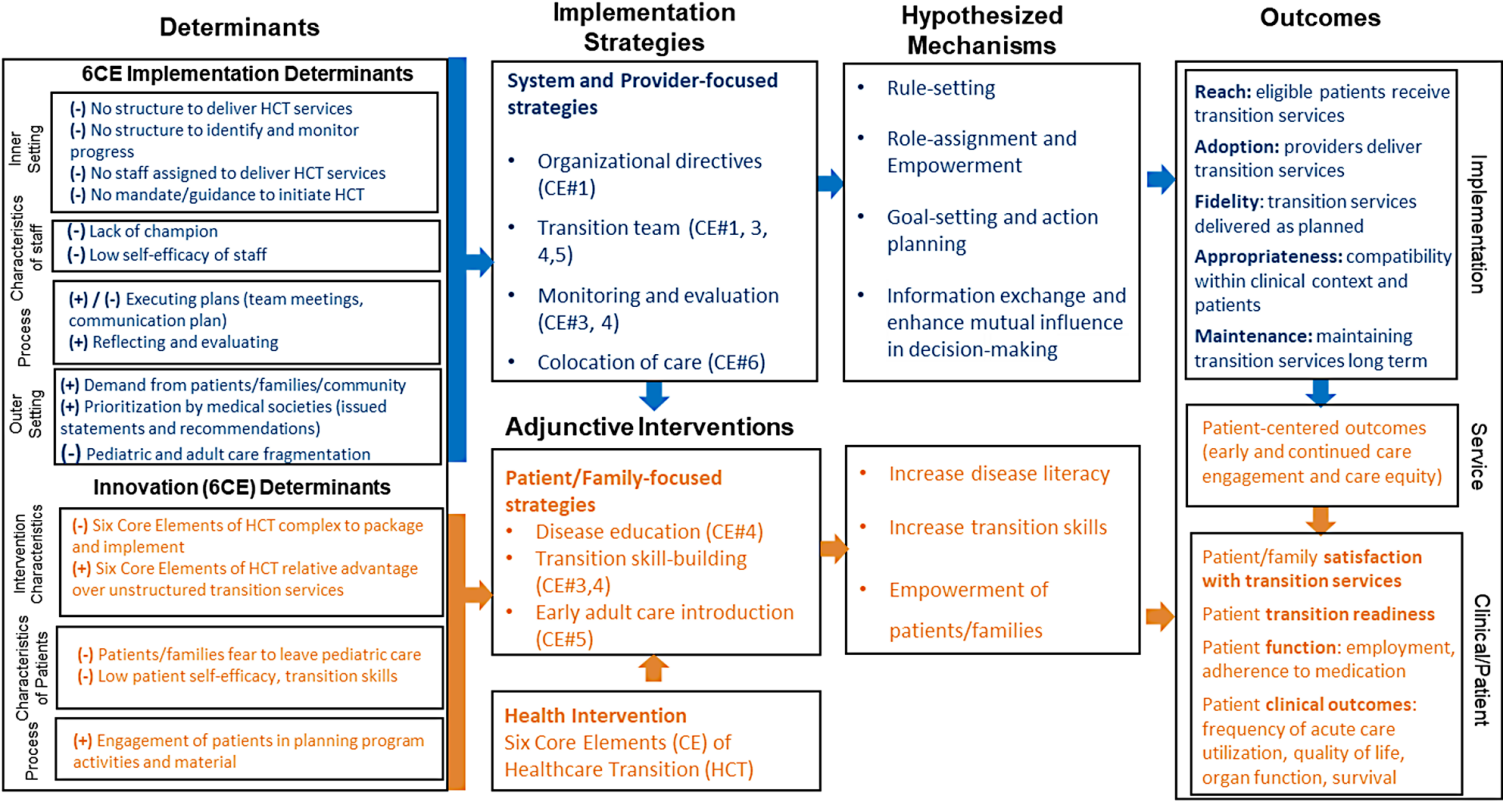
Implementation research logic model for the transition to adult care program

We began the IRLM by listing the determinants within the following domains: intervention characteristics, inner setting, outer setting, characteristics of the individuals, and process (Fig. 1). During the process of outlining implementation strategies for each of the 6CE, we identified that some program strategies employed behavioral change methods intended to increase patients’ self-efficacy and knowledge, to promote adherence to HCT services, which we then classified as adjunctive interventions. Adjunctive interventions are change methods that target recipients (e.g., patients, program participants) of a health intervention and are designed to increase recipients’ motivation, self-efficacy, or capacity to adhere to or comply with the health intervention [84]. We identified three adjunctive interventions: (1) “Disease education,” which entails providing patients with clinical sessions outlining SCD and SCD management; (2) “Transition skill-building,” which entails teaching adolescents skills around health navigation and healthcare management (e.g., scheduling appointments in the adult setting), and (3) “Early Adult Care Introduction” which involves patients visiting the adult provider’s offices and staff before they leave pediatric care (Supplemental Table 1). This distinction between implementation strategies and adjunctive interventions is important as it highlights the complexity of HCT that requires both implementation strategies and adjunctive interventions to enact the 6CE at both the staff and patient/caregiver levels. Once all implementation strategies and adjunctive interventions were identified and mapped, we hypothesized the mechanisms by which the implementation strategies were expected to work, based on a literature review and discussions with the HCT team members.

### Theories that informed the mechanism of the implementation strategies

We identified six theories that informed our proposed theory of mechanisms of HCT; see also Table 1 and Supplemental Table 1.

#### Institutional theory

Institutional theory conceptualizes the role of actors (i.e., individuals, organizations, or national entities) as both affected by and affecting the organizational environment [85–87]. We used this theory to help us understand how various aspects, such as pressures from regulatory bodies and professional norms (e.g., organizational directives regarding HCT transition), affect the adoption of new practices. In the context of HCT, Institutional Theory reminds us about the importance of clear organizational policies (e.g., when to start HCT) for the success of the HCT process.

#### Transaction cost theory and resource dependence theory

Because SCD has different local care systems (i.e., care is provided by various institutions within the system), we need to recognize the importance of exchanges within organizations (e.g., pediatric and adult settings) [88–91]. We can use transactional theory to examine how to establish the co-location of services between pediatric and adult settings. For example, we realized that we may need to emphasize the importance of contracting or payment for services in the context of co-locating HCT activities [92]. Similarly, transaction theory and resource-dependent theories can inform resource allocation to each HCT activity, both in the pediatric and adult settings, depending on existing and/or available resources.

#### Contingency theory

Reminds us that the uncertainty of tasks can impact the success of programs. Uncertainty arises from the gap between the information needed to perform a task and the information available (e.g., when to start HCT and what steps are needed to support adolescents during HCT). Contingency theory and goal-setting theories remind us of the importance of having a clear goal, motivating workers to achieve it, and providing the necessary resources to accomplish the goal [93]. 

#### Social identity

The literature on social identity posits that people tend to categorize themselves according to various roles, such as those in healthcare [94]. For example, shifting one profession in terms of occupational role (e.g., a pediatric hematologist focused only on general SCD care of children shifts to working on HCT specifically) can influence nearby professions (e.g., nurses, social workers). Here, the theory reminds us that examining how collaboration (or competition) among professionals can affect the uptake of HCT practices is important.

#### Networks and organizational change

We draw on two theories of complex systems to inform the program’s design. First, social network theory emphasizes the importance of examining the patterns and quality of information being shared between providers. Network effectiveness, both as a process and as an outcome, affects healthcare delivery [95]. Second, elements of the program highlight components of complexity theory; it is essential to examine how top-down approaches (e.g., establishing HCT guidelines) must be matched with supporting professionals to engage in bottom-up changes (e.g., re-aligning roles and actions during the organization’s restructuring).

### Step 3. Isolate the mechanism of each implementation strategy

**Fig. 2:**
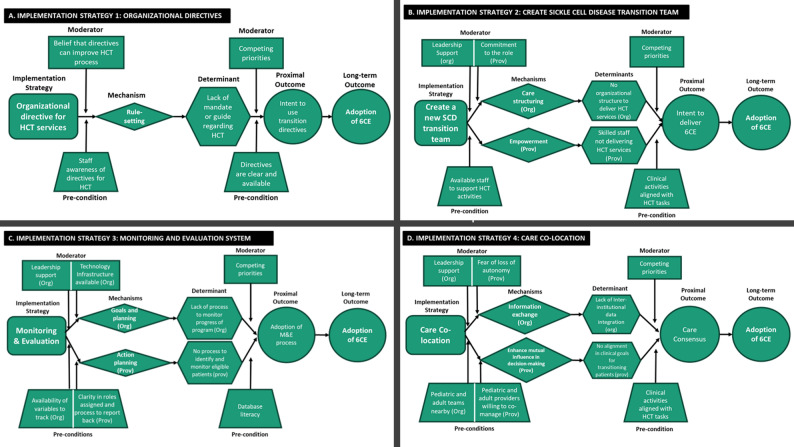
Causal pathway diagrams for the St. Jude HCT Program. Four implementation strategies were identified: (**A**) Organizational directives, (**B**) Create a new sickle cell disease transition team, (**C**) Monitoring and Evaluation, and (**D**) Co-location of care. Their pre-conditions and moderators are outlined by target level (provider or organization) as well as their mechanisms and outcomes (proximal and distal)

Next, the transition team generated a list of outcomes, preconditions for mechanism activation, proximal outcomes, and distal outcomes for each strategy (Supplemental Table 2). This exercise allowed us to realize that the same strategy could have more than one mechanism and that this depended on the target level of the intervention (provider versus organization). For instance, the SCD transition team strategy stimulates structuring care at the organizational level while promoting empowerment at the provider level; care co-location activates information exchange between organizations while enhancing mutual influence in provider decision-making. To better visualize the mechanisms of action, we developed causal pathway diagrams for each implementation strategy. Figure 2 illustrates the putative mechanisms that are expected to target the determinant, as outlined, along with precondition moderators that are expected to be relevant for mechanism activation (both at the institution and provider levels), proximal outcomes that are expected to indicate mechanism activation, and distal outcomes. Detailing the variables that influence the function of the strategies, including pre-conditions and moderators, as well as the outcomes, allowed us to conceptualize the pathway through which each strategy worked to increase the adoption of HCT practices for adolescents and young adults with SCD.

### Step 4. Visualizing systems of care

**Fig. 3:**
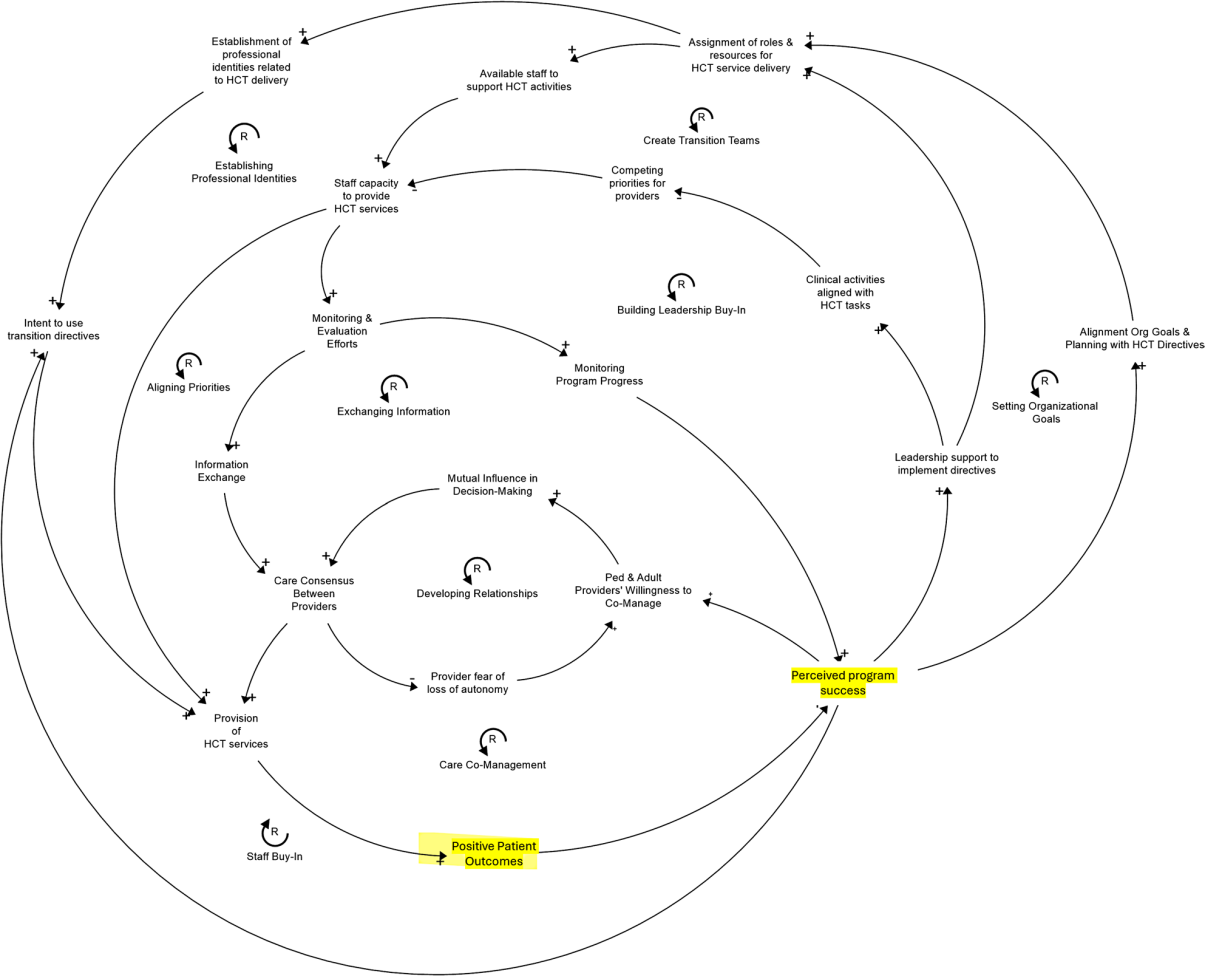
Causal looping diagram for the St. Jude HCT Program. A main CLD depicts the multiple reinforcing loops that encompass the main mechanisms where the implementation strategies enact 6CE, i.e., allow the provision of HCT services. Perceived program success, as informed by the positive patient outcomes (both highlighted in yellow), arose as an important mediating factor not previously identified through the causal pathway diagrams.

Finally, a CLD was developed to illustrate the integration of the four implementation strategies across multiple system components, thereby conveying that the implementation strategies do not impact the system in isolation (Fig. 3). The identified implementation strategies impact the program through multiple interconnected mechanisms represented through reinforcement feedback loops. For example, developing new staff teams was considered in terms of having staff available, role assignments, and structures to deliver HCT, amongst others. This allowed for a more cohesive assessment of how the system operated and can continue to develop over time. The CLD uncovered another key mediating factor, not previously identified by the causal pathway diagrams, namely, the perceived program success, as informed by the positive patient outcomes (e.g., retention in care) (Fig. 3). In the results below, we detail three key overarching processes revealed by the CLD: developing capacity for program delivery, enabling successful collaboration between providers, and maintaining leadership support. These three processes appear foundational and are hypothesized to be necessary for implementing the 6CE for the St. Jude HCT Program.

### 1 - Developing capacity for program delivery (Fig. 4)

Successful service delivery requires capable and effective staff. In the case of the St. Jude HCT program, one of the core implementation strategies involves developing this team (SCD transition team), which defines a new identity for the staff members, a key step in fostering intent in delivering HCT services. However, this strategy does not exist in isolation and is not sufficient to build capacity for program delivery. The mechanisms for change, care structuring, and empowerment are all impacted by Implementation Strategy 1 (organizational directives), which establishes and carries out organizational directives. This is due to the idea that alignment with organizational goals is necessary to maintain the operation of the transition team. The clinical mandates may result in care structuring through increased structural support and the creation of roles for HCT services provision (Loop: Create Transition Team). Creating roles and establishing a professional identity associated with HCT delivery can lead to greater staff commitment to executing organizational directives through the mechanism of empowerment (Loop: Empowerment).

Organizational directives for HCT priorities can also impact key moderators for transition team development. Directives that help align clinical workflows with HCT delivery lead to fewer competing priorities, contributing to staff capacity to deliver the program (Loop: Aligning Priorities). In turn, a well-supported team can help reinforce organizational directives. Over time, successful program delivery and positive patient outcomes can further increase staff buy-in and intent to use transition directives, the proximal outcome for Implementation Strategy 1, SCD transition team (Loop: Staff Buy-In).

**Fig. 4:**
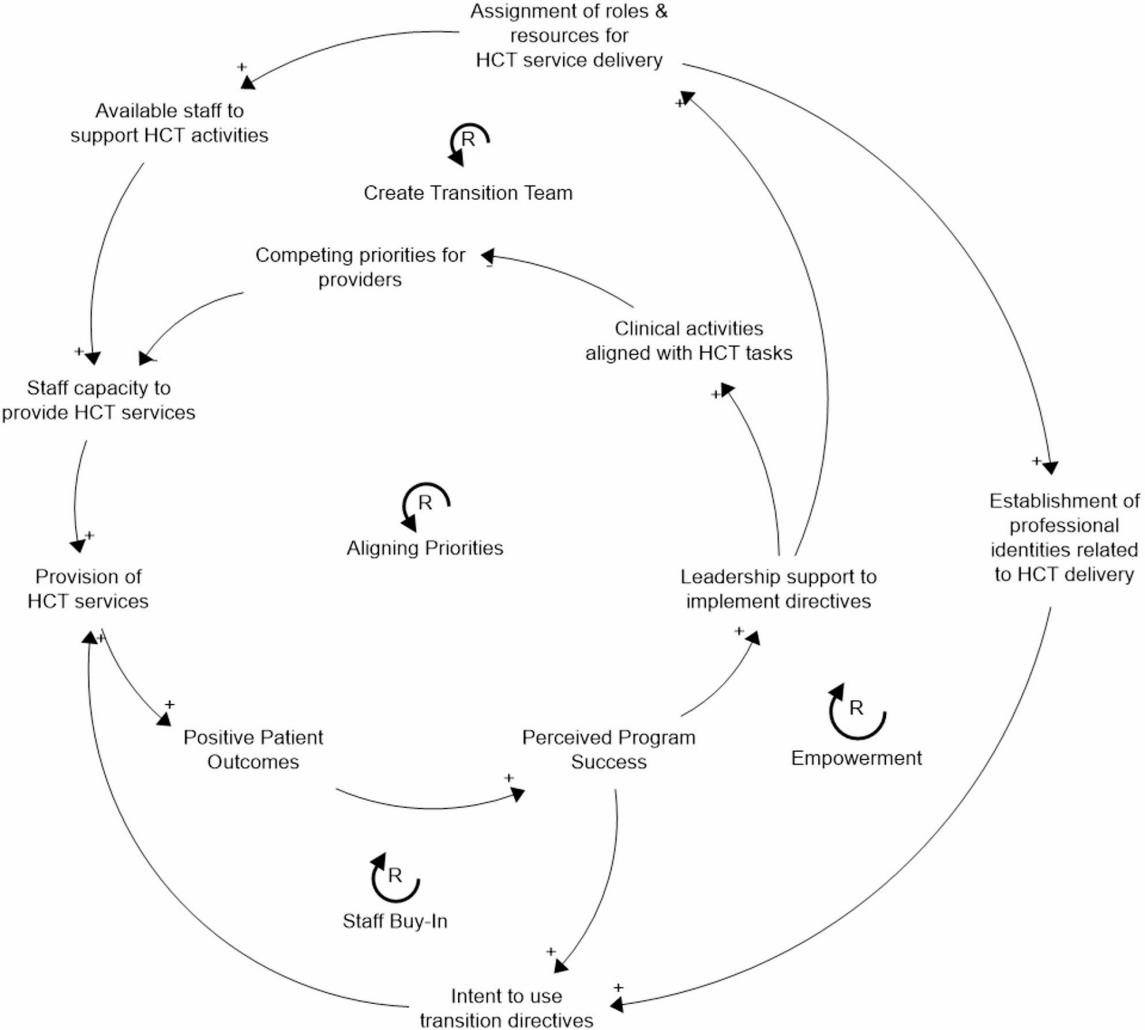
Capacity development loop. Implementation Strategy 1 (organizational directives for health care transition) impacts Implementation Strategy 2 (create SCD transition team) by reinforcing the team operations and supporting its ongoing function

### 2- Enabling successful collaboration between providers (Fig. 5)

While individual teams can deliver HCT services, Implementation Strategy 4 (care co-location) plays a crucial role in facilitating cross-team collaboration between adult and pediatric providers. However, cross-team collaboration extends beyond care co-location to intersect with other components of the system. As with the development of care teams, organizational directives and leadership support both help reduce competing priorities and increase overall capacity for service provision. Implementation Strategy 3 (monitoring systems) and Implementation Strategy 1 (organizational directives) can help enable successful collaboration between adult and pediatric providers while delivering HCT services. Identifying and monitoring eligible patients facilitates the mechanism of information exchange by providing the necessary patient data to arrive at care consensus (Loop: Exchanging Information). In addition, while enhancing mutual influence in decision-making is partly dependent on provider-provider interaction (Loop: Developing Relationships), demonstrating success of the program over time through program monitoring and evaluation can help increase providers’ buy-in to the program and willingness to co-manage (Loop: Care Co-Management).

**Fig. 5:**
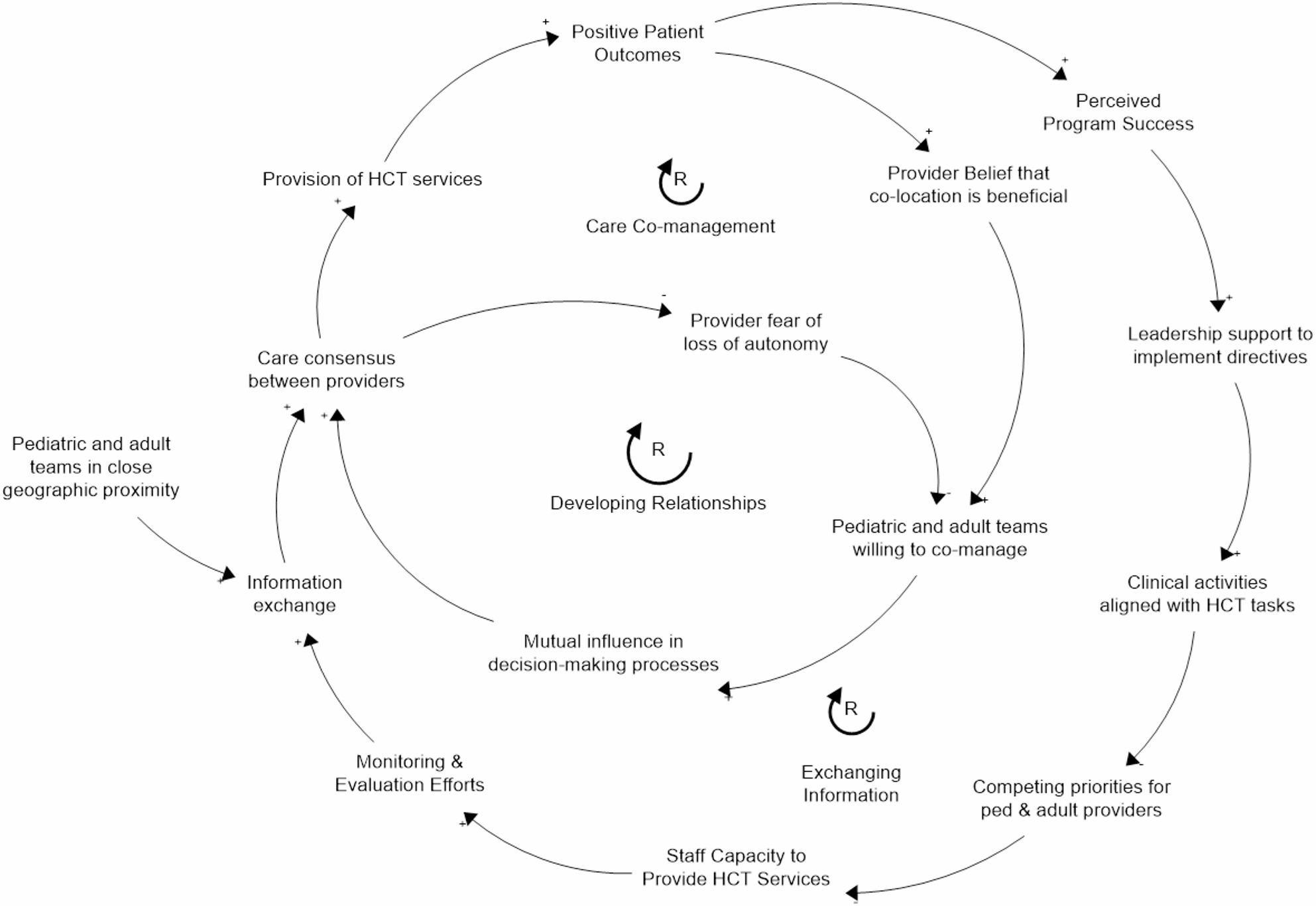
Collaboration process. Implementation Strategy 4, Co-location, does not occur in isolation, as Implementation Strategy 3, Monitoring systems, and Implementation Strategy 1, organizational directives, enable successful collaboration between adult and pediatric providers, thus promoting exchange of information, building relationships, and supporting co-management, thus fostering the delivery of HCT services

### 3- Maintaining Leadership Support (Fig. 6)

Leadership support is a key moderator across three of the four implementation strategies. Strong, supportive leadership is essential for allocating resources for program delivery, devoting time to monitoring and evaluation efforts, and permitting care co-location. However, leadership support is not just an input, as it also evolves in response to program success. Just as leadership support fosters capacity building and provider collaboration for HCT service delivery, effective service delivery reinforces and sustains leadership support, creating a mutually reinforcing cycle. Monitoring and evaluation can also help maintain leadership support through communication of program evaluation results (i.e., positive patient outcomes). Even when a program leads to positive patient outcomes, there can be a disconnect between those outcomes and leadership’s perception of the program. Evaluation allows staff members to clearly demonstrate and communicate program value (Loop: Building Leadership Buy-In). Sustaining leadership support ensures future organizational goals and planning are aligned with HCT service delivery and sufficient resources are allocated for the program.

These pathways may not all function at the same time or to the same degree. Organizational Directives may dictate that staff are assigned to HCT service delivery, but staff members may not feel empowered within their role or committed to executing transition directives. In other instances, staff may be committed to the program but unable to deliver services due to competing priorities. Similarly, feedback loops can vary in strength and speed, introducing delays between program launch and measurable patient outcomes. During this interim period, strong provider relationships or empowering staff through establishing professional identities may play a more important role than overarching outcomes in facilitating program delivery.

**Fig. 6:**
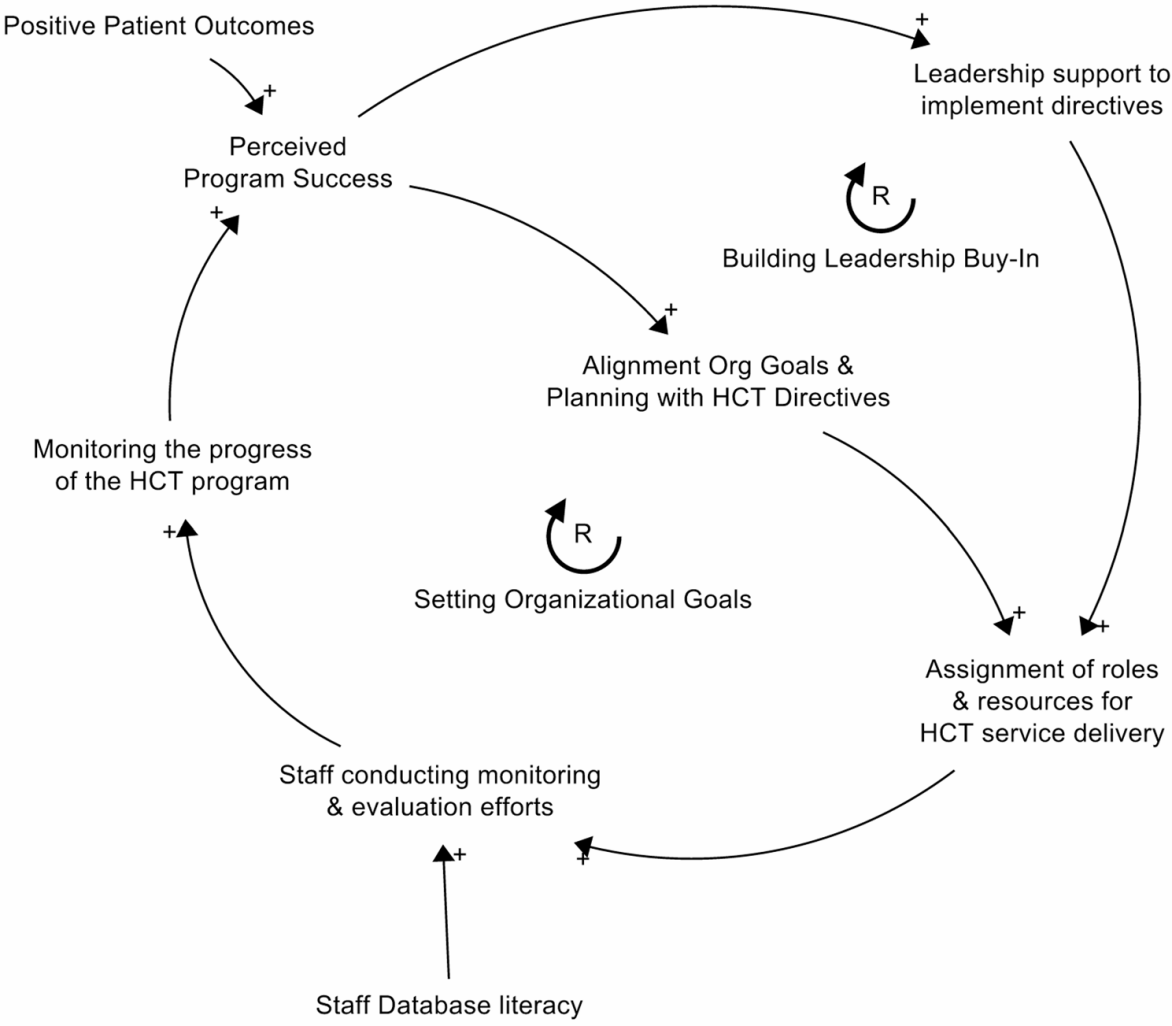
Leadership Buy-In. Leadership support evolves in response to program success. As leadership support fosters capacity building and provider collaboration for HCT service delivery, effective service delivery reinforces and sustains leadership support, creating a mutually reinforcing cycle that sustains the transition program’s operations

## Discussion

While recognized as important and necessary, HCT programs are complex to implement. Through a 4-step process, we began to disentangle the mechanisms of action of a HCT program for SCD that has implemented the 6CE. We identified four implementation strategies: organizational directives, SCD transition team, monitoring and evaluation, and care co-location, that together enact the 6CE and sustain program functionality. Validating the complexity of the HCT program, we showed in this case study that these strategies functioned through different hypothesized mechanisms, both at the organization and provider levels, sometimes, with more than one mechanism per strategy, depending on the target level.

Our methodology for identifying the strategies, hypothesizing their mechanism, and theorizing how they function together follows a similar recommended sequence of steps [96], but uses a retrospective (post-implementation) approach. The key moderating and mediating factors (i.e., the contextual factors) that cut across implementation strategies can help programs prioritize efforts in managing the key linchpins to success in the system. As programs take shape, it is essential to continuously demonstrate their impact to reinforce their value and increase their likelihood of sustainability.

The goal of this study was to develop a roadmap for implementing HCT programs. Part of the challenge in replicating transition programs that use 6CE is its complexity. Our roadmap based on the St. Jude HCT program, a successful program for HCT, has the goal of being at the same time context-dependent (e.g., the number of staff in the HCT care team, the specific tasks for each team member) while also generalizable. By providing a clear description of how dynamic, interrelated, and multi-level the implementation process of the 6CE is, in addition to the fact that different strategies have different time points [97], we hope scholars can engage in replicability of the 6CE program by testing the discrete strategies identified here in different contexts [39]. We hope that the hypothesized mechanisms of the implementation strategies used to implement one HCT program function as a proposed theory for how the strategies work and can be empirically tested, thus confirming or refuting the hypothesized mechanistic assumptions made here. Our CLD also informs future intervention selection and adaptation, as one can consider how the model suggests where and how we would expect to see change. For instance, co-location of providers in the same geographical space may not be possible in certain settings (due to staff availability or cost); however, selecting a different strategy that increases information exchange across institutions and enhances mutual decision-making among providers could achieve the same goal of increasing the adoption of HCT services. In other words, the same mechanism can be achieved through different implementation strategies, depending on the critical contextual factors, as demonstrated here. Understanding the mechanisms of strategies and how contextual factors impact their mechanisms will improve the transportability of implementation to different settings and contexts, thus increasing generalizability [98]. 

A key aspect of our findings is the timing of the strategies. Other scholars have noted the importance of temporality and ordering of strategies in the deployment of complex interventions (e.g., Chamberlain et. al [99] and Bunger et. al [100]). Here, we show, for example, that the HCT process is initiated by an organizational directive, which may have a one-time point in the process. However, other strategies, such as the SCD transition team and the monitoring and evaluation, are embedded across the HCT process. Alternatively, care co-location serves as a vehicle for information exchange and the development of care consensus between providers, which in turn supports the provision of HCT services.

Understanding the underlying causal connections between strategies and outcomes can facilitate adaptation, particularly when implementing the complete intervention strategy is not feasible, such as due to space constraints. In such cases, providing alternative pathways for information exchange may be a viable option. Furthermore, understanding the connections and feedback loops within the system can help identify why a strategy is *not* working. In the example of care co-location, providers may be in the same place and yet still not have enough time for shared care planning because of competing priorities, which, in turn, is affected by leadership buy-in for implementing organizational directives. The careful evaluation of the different components of each strategy, along with examination of their relationship, reminds us that solving an issue with one implementation strategy may require activation of another. Additionally, evaluation along the implementation process may be important. As the system changes in response to implementation efforts, things that were not feasible when the program started may become more feasible as success starts to show and momentum builds. Conversely, programs that do not consider how a system responds to change may unintentionally encounter roadblocks created by their own implementation efforts. CLDs allow us to dynamically evaluate ongoing changes in the system, positioning the team to more nimbly adapt to external pressure for change.

The CLD reveals key processes that contribute to the long-term sustainability of the program. The St. Jude HCT program, which has operated continuously for over 15 years and served more than 700 youth with SCD [59], exemplifies these dynamics. Its sustained operation is underpinned by ongoing capacity development, driven by consistent leadership support and strong staff collaboration. These foundational processes appear essential for maintaining conditional organizational commitment and securing continued investment in the program.

### Strengths and limitations

While following a rigorous methodology to identify strategies that are continuously used to implement the 6CE in one program, and considering the participatory involvement of staff and advocates, our method was retrospective and may have left out relevant data. However, we followed rigorous analytical methods to retrospectively inquire about team function, activities, and informed our identification of strategies and their mechanisms through theory, thereby strengthening our findings. Our methodology for understanding the mechanism and implementation of HCT may be translatable to other chronic conditions, such as type 1 diabetes or cystic fibrosis. Another limitation of identifying key elements of the St. Jude HCT program is the high-resource setting in which these diagrams were developed as a case study. Nevertheless, we believe that the key mechanisms identified are not unique to our institution. As we study different environments, contextual differences may diminish, as problems may reach saturation due to similar barriers and enablers. Our long-term goal is to develop a methodology that facilitates the adaptation of strategies to implement 6CE for SCD in other settings, particularly those with limited resources. We plan to apply this methodology in a middle-income country, where resources are not as plentiful but where the need and desire to structure HCT services are high.

## Conclusions

Through a four-step method, we identified four key implementation strategies that enact the 6CE: establishing an organizational mandate, forming a dedicated SCD transition team, instituting monitoring and evaluation processes, and co-locating care. Furthermore, we identified three underlying key processes: capacity development, staff collaboration, and leadership buy-in, which not only facilitated the implementation of the 6CE but also appeared to be central to the program’s long-term sustainability. This work offers a roadmap for identifying and integrating effective strategies to implement HCT programs, suggesting the mechanisms through which they operate and interact to support the 6CE framework for SCD. We theorize the contextual factors necessary for these strategies to be effective, enhancing their adaptability and potential for translation across diverse settings and populations. We propose that this methodology be further used when retrospectively studying complex clinical programs and potentially adapted for other chronic conditions within the healthcare system. We hope this methodology will facilitate the future implementation of HCT programs by providing a better understanding of the necessary and sufficient strategies, mechanisms, and processes for HCT service delivery.

## Supplementary Information

Below is the link to the electronic supplementary material.

Supplementary Material 1

## Data Availability

The datasets used and/or analyzed during the current study are available from the corresponding author on reasonable request.

## Abbreviations

6CE: 6 Core Elements
SCD: Sickle cell disease
HCT: Health care transition
CLD: Causal loop diagrams
IRLM: Implementation research logic model
