# Supplementary material for: Mechanism-informed transition from pediatric to adult care in sickle cell disease: a case study
Source: BMC Health Serv Res. 2026 Mar 13;26:563. doi: 10.1186/s12913-026-14312-9 (PMC13101270; doi:10.1186/s12913-026-14312-9)
Supplement: Supplementary file 1 — Supplementary Material 1 [file 12913_2026_14312_MOESM1_ESM.docx]

**Supplemental Table 1.** Description of the components of the adjunct interventions

| **Determinant** | **Adjunct Intervention** | **Definition** | **Action** | **Actor (who delivers the strategy)** | **Action Target** | **Dose (frequency)** | **Outcomes** | **Theories informing** |
| --- | --- | --- | --- | --- | --- | --- | --- | --- |
| Low knowledge of the disease to support self-management | Disease education (disease literacy building) | Provision of educational sessions in the clinic and online. Educational material delivered | Receipt and engagement with education materials | SCD Transition staff | Adolescents and young adults | Every 6 months | Increase in patient knowledge in self-management | Social cognitive theory(101, 102)  Health belief model(103)  COM-B(70) (Capability) |
| Low self-efficacy in transition skills | Transition skill-building | Training of transition skills offered to adolescents (e.g., scheduling appointments) | Receipt and engagement with transition skill education and materials | SCD Transition staff | Adolescents and young adults | Once | Increase in patient skills in navigating the adult care system | Social cognitive theory(101, 102)  Health belief model(103)  COM-B(70) (Skills) |
| Families invested in maintaining care continuity | Early adult care introduction | Visit of adult providers’ offices and staff before patients leave pediatric care | Increase awareness and comfort with future providers.  Peer young adults with SCD share their experiences through testimonials about the disease and engagement with healthcare. | SCD Transition staff | Adolescents and caregivers | Once | Increase patient comfort and empowerment | Empowerment theory(104, 105)  COM-B (Motivation) |

**Note:** Adjunct interventions are specified according to Proctor et al. classification(41). SCD: sickle cell disease COM-B: capability, opportunity, motivation, and behavior change model.

**Supplemental Table 2.** Pathway of implementation strategies

| **Strategy** | **Precondition for Mechanism Activation** | **Moderator**  **For the mechanism of action** | **Mechanism of action** | **Precondition for Proximal Outcome** | **Moderator for proximal outcome** | **Proximal outcome** | **Distal outcome** |
| --- | --- | --- | --- | --- | --- | --- | --- |
| Organizational Directives | Staff are aware that there are directives for health care transition (HCT) | Believe that the directives can improve HCT | Rule-setting | Directives are clear and available to staff members (organization and provider levels) | Familiarity with directives (provider level)  Leadership support to implement directives (organizational level) | Adoption of transition directives | Directive-concordant HCT practices |
| SCD transition team | Available staff to support HCT activities  Clear roles among team members | Leadership support  Commitment to role | Care structuring (Organization)  Empowerment (Provider) | Clinical activities aligned with HCT tasks | Competing priorities | Intent to deliver HCT services | Adoption of HCT practices |
| Monitoring and Evaluation (M&E) | Availability of a list of variables to track  Clarity in roles assigned (e.g., who tracks)  Clarity on a process to report back (who analyzed the data, when, how often, reports to whom) | Leadership support  Infrastructure available for monitoring (e.g., computers, database) | Goals and planning (Organization)  Action planning (Provider) | Database literacy | Competing priorities | Adoption of M&E process (staff enter data and use the database to monitor transition outcomes) | Adoption of HCT practices |
| Pediatric and adult care co-location | Pediatric and adult teams in close geographic proximity  Pediatric and adult teams are willing to co-manage | Leadership support  Provider's fear of loss of autonomy  Belief (from pediatric and adult providers) that co-location is beneficial | Information exchange (Organization)  Enhance mutual influence in decision-making and clinical practice (Provider) | Clinical activities aligned with HCT tasks | Competing priorities from both pediatric and adult providers | Consensus of care between providers (development of a care pathway)  Positive relationship between pediatric and adult providers | Adoption of HCT practices |
